# A review of contemporary autonomy: ethics, authority, and the fragility of the late modern subject

**DOI:** 10.1007/s11019-026-10351-5

**Published:** 2026-04-20

**Authors:** Luis Madeira, Cíntia Águas

**Affiliations:** 1 Faculdade de Medicina, Universidade de Lisboa, Av. Prof. Egas Moniz, Lisboa, 1649-035 Portugal; 2 Hospital CUF Descobertas, Lisboa, Portugal

**Keywords:** Autonomy, Late modernity, Ethical maturity, Duties, Symbolic authority

## Abstract

This conceptual review examines how autonomy became the dominant moral grammar of late modern Western societies and how its expansion reshapes subjectivity, institutions, public life, and healthcare practice. A genealogy traces autonomy from Enlightenment duty bound self legislation and civic self rule, through liberal non interference, to later forms shaped by expressive individualism, therapeutic culture, and recognition based claims. The paper argues that autonomy’s normative achievements remain decisive, yet its cultural absolutization can generate a fragile subject who is highly expressive but insufficiently equipped for sustained responsibility. Five linked pathologies are analysed: autonomy as entitlement and boundary protection, transgression emptied by indeterminate norms, opinion transformed into performative positioning under hyperpluralism and platform surveillance, symbolic authority eroded across education and the professions, and duties devalued within a rights centred moral economy. In response, the paper proposes a reconstructive framework that redefines autonomy as ethical maturity, a formed capability sustained by responsibility, legitimate authority, deliberative practices of opinion, and duty. Particular attention is given to philosophy of medicine, where patient autonomy and professional autonomy illuminate the difference between supported agency and consumerist choice. Methodologically, the article combines genealogical reconstruction, phenomenological interpretation, and a purposive interdisciplinary synthesis of sociology, developmental psychology, philosophy of medicine, moral psychology, and media studies. Implications are sketched for education, healthcare, professional trust, and platform governance, with emphasis on proportional accountability and repair.

## Introduction

In the moral and political imagination of modernity within Western thinking, autonomy stands as the foundational value. From Kantian ethics to liberal democracies, the sovereign individual, rational, self-directed, and free, has become the primary unit of ethical and legal reasoning (Kant and Gregor 1998; Mill 2010) The concept of autonomy has progressively expanded, not only securing rights and freedoms, but also with social impact, shaping identity, education, healthcare, consumer individualism, and interpersonal relations. Yet, as autonomy reaches its cultural zenith, a paradox emerges: the contemporary subject appears maximally free but existentially fragile, lacking the developmental, symbolic, and social structures necessary to sustain meaningful selfhood.

If we contextualise the rise of autonomy historically we find in the enlightenment philosophy the emergence of moral autonomy as the capacity to legislate one’s own maxims through reason, thus opposing external heteronomy (Kant and Gregor 1998; Schneewind 2008). Political liberalism translated this into legal frameworks where individuals enjoy freedom from coercion, provided they do not harm others (Mill 2010). In the 20th century, this model was expanded in two directions. First, through expressive individualism, which made self-realization the ultimate moral goal (Taylor 1989; Bellah 2002), equating autonomy with the right to define one’s identity and aspirations based on inner feelings and intuitions, which in turn claim to be externally acknowledged and protected. Second, through the therapeutic turn, which reframed autonomy in terms of emotional well-being, self-management, and authenticity (Rieff 1987; Illouz 2008), where choices deemed as “good” are those that promote well-being and self-esteem, often guided by psychological support. While these expansions enabled greater inclusion and recognition, they also untethered autonomy from embeddedness, relationality, and the moral weight of shared traditions (MacIntyre 2007). As symbolic boundaries blur, transgression becomes impossible, not because norms are too rigid, but because they have become indeterminate. Classical and developmental accounts view transgression as essential to maturation. The child must confront limits to develop autonomy, responsibility, and recognition of the other(Erikson 1994; Winnicott 2018). In contrast, the contemporary adolescent often grows in a permissive environment where normativity is either privatized or externalized, which in our liquid contemporary society it increases the sense of disorientation (Lasch 1978; Bauman 2013). This cultural shift has challenged adult subjectivity, as the limit probing process integral to forming adult autonomy is replaced by fragile performativity.

The loss of normative structures also contributes to the paralysis of opinion. In traditional societies, the repression of dissent limited plurality. Today, the opposite occurs: difference is overvalued to the point of fragilizing judgment itself. In *hyperpluralistic* settings, affirming any strong opinion risks being seen as invalidating others, leading to expressive inhibition or alignment with identity politics (Han 2018). Rather than fostering deliberation, this environment fuels autocensorship and a fear of *“cancel culture”*, especially in digital arenas dominated by surveillance and performativity (Sunstein 1999; Furedi 2013). Compounding this is the erosion of authority across domains, family, school, institutions. Authority, when just, is not merely domination but a formative structure enabling learning, respect, and symbolic transmission (Arendt 1993; Sennett 2006). Its decline has produced institutional distrust, epistemic fragmentation, and pedagogical uncertainty. In education, for example, teachers often struggle to assert their roles as guides without being perceived as oppressive, while students increasingly appeal to identity politics as moral capital (Brighouse 1998; Lenta 2016).

Finally, the expansion of the rights discourse has occluded the moral language of duties. In the human rights framework, duties are often instrumental, i.e., obligations that fall upon third parties and institutions duties to either protect, respect or fulfil rights that are universally acknowledged to all human beings, even if sometimes complemented by specific rights pertaining to certain groups. In this sense, human rights are rarely seen as ethical achievements that build character and cohesion (Jonas 1984). The absence of a dutybound ethos has consequences for public life: civic engagement wanes, professional obligations are seen as burdens, and collective projects are fragmented (Etzioni 2013; Tronto 2013). In work, education, and family life, duties are no longer valorised but negotiated, contributing to the erosion of shared purpose and institutional trust (Sandel 2020).

This essay examines these interlinked dynamics through a multidisciplinary lens, drawing from philosophy, cultural criticism, moral psychology, sociology, and philosophy of medicine. It critically appraises the dominance of autonomy in late modern societies, acknowledging its historical importance and normative achievements while exploring the pathologies emerging from its absolutization. In particular, the paper interrogates five interlinked phenomena: (1) the hegemony of autonomy, (2) the loss of transgression, (3) the paralysis of opinion, (4) the collapse of symbolic authority, and (5) the devaluation of duties in a rights focused moral economy. The argument also bears directly on medicine and mental healthcare. In clinical ethics, autonomy has been indispensable for resisting paternalism and grounding informed consent, yet clinical practice shows that autonomy is rarely exercised as isolated preference satisfaction. Patient decision making is relational, dependent on communication, trust, interpretive support, and professional judgment, while clinicians require a form of professional autonomy ordered to fiduciary responsibility rather than consumer demand (O’Neill 2002; Entwistle et al. 2010; Beauchamp and Childress^2019^; Montgomery 2023). The aim is therefore not to dismiss autonomy but to resituate it within a broader moral ecology that includes transgression, opinion, authority, duty, and the relational conditions under which autonomy becomes sustainable.

## Methodology

This article is a conceptual essay and this section clarifies analytical approach, argumentative procedure, and source selection rather than presenting a reproducible dataset. The first step is genealogical reconstruction. Key moments in the meaning of autonomy are traced from Enlightenment moral and political thought, through liberal jurisprudence, to contemporary therapeutic, recognitional, and platform mediated forms. The genealogical thread follows authors, concepts, and institutional settings selected because they mark identifiable shifts in the normative grammar of autonomy. The second step is phenomenological interpretation. Autonomy is read as a lived structure of agency, dependence, vulnerability, temporality, and answerability, with attention to how subjects experience freedom, limits, obligation, recognition, and authority. The third step is an interdisciplinary interpretive synthesis that tests the plausibility of the philosophical diagnosis against relevant work in sociology, developmental psychology, political theory, media studies, and philosophy of medicine. Sources were selected purposively according to three criteria: canonical importance for the concept under discussion, contemporary relevance to late modern institutions and digital culture, and direct usefulness for clarifying the paper’s core claims. Empirical studies are therefore used as orienting and constraining materials, not as a dataset from which causal claims are inferred.

## Discussion

### A genealogy of autonomy: from enlightenment to therapeutic culture

Autonomy, understood as the capacity to govern oneself according to rational or authentic principles, has undergone a major historical transformation. From its Enlightenment roots as a moral and political ideal tied to self-legislation and civic formation, autonomy has expanded into a cultural imperative of self-expression, emotional authenticity, and recognition seeking. We aim first at presenting the trajectory from Kant and Rousseau through liberal individualism and the harm principle, toward expressive individualism and therapeutic culture, where autonomy is increasingly measured by felt coherence, personal boundaries, and the demand for validation.

For Immanuel Kant, autonomy names the rational will’s capacity to give itself the moral law. It is not mere choice but duty bound self-governance, acting on maxims that can be willed as universal (Korsgaard et al. 1996; Kant and Gregor 1998). Autonomy here is inseparable from obligation, the sovereign subject is constituted by the binding force of reason rather than by preference. Jean Jacques Rousseau uses a similar view of autonomy into the sociopolitical domain. Freedom is secured not by private independence alone but by participation in the general will, a civic form that binds individuals to a shared normative project (Neuhouser 1993; Rousseau 1996). Kant and Rousseau thus offer two complementary foundations: moral self-rule through universal obligation, and civic self-rule through collective authorship, both presupposing a thick normative order that forms the subject. John Stuart Mill recasts autonomy within liberalism as a protection against coercion and interference. His harm principle establishes a juridical boundary, as power is justified only to prevent harm to others, and individuality is valued as an engine of development and social progress (Gray 2006; Mill 2010). Autonomy becomes increasingly identified with negative liberty and personal experimentation, grounded less in duty than in the legitimacy of plural life plans. This shift proves decisive for later rights-based frameworks, where autonomy is often treated as a formal entitlement rather than a formed capacity.

In the 20th century, autonomy expands again, from political status to cultural psychology. Expressive individualism makes authenticity a central moral ideal - the self is expected to realize its inner truth and display coherence between feeling and identity (Taylor 1989; Bellah 2002). The therapeutic turn strengthens this reorientation. Psychotherapy, consumer culture, and self-help norms convert autonomy into a project of emotional management, self-narration, and boundary work, where wellbeing becomes a primary measure of legitimacy (Rieff 1987; Cushman 1990; Illouz 2008). In this setting, the autonomous subject is not only free to choose but compelled to choose a life that signifies authenticity and protects psychic integrity. A further step is the moralization of identity and vulnerability. Under recognition based frameworks, autonomy becomes increasingly articulated through claims to visibility, protection from symbolic harm, and the validation of lived experience as moral standing (Honneth and Honneth 1996; Fraser 2000; Brown 2008). Moral authority is often grounded in the credibility of suffering, trauma, or marginalization, and autonomy is increasingly negotiated in affective registers rather than through shared criteria of justification (McNay 2008; Fassin et al. 2009). This transformation has corrected important exclusions, yet it also shifts the locus of normativity, from duties and common practices toward recognition, safety, and subjective integrity. This genealogy clarifies the pivot of the present argument. Autonomy’s modern achievements are substantial, but its late modern expansion tends to detach freedom from formative limits, shared standards of judgment, and the durable institutions that transmit responsibility. The result is not the disappearance of normativity but its relocation into unstable moral economies, therapeutic validation, and platform mediated visibility. It is at this point that the five pathologies analysed in the following sections become legible: the hegemony of autonomy as a dominant moral grammar; the loss of transgression as a developmental and civic mechanism; the paralysis of opinion under hyperpluralism and performative speech; the collapse of symbolic authority and institutional trust; and the devaluation of duties in a rights-centred moral economy.

### Autonomy in healthcare and bioethics: from informed consent to relational agency

In clinical ethics, respect for autonomy emerged as a corrective to paternalism and remains indispensable for informed consent, refusal of treatment, and bodily integrity and self-governance. Yet illness is not an ordinary choice environment. Patients deliberate under fear, uncertainty, asymmetries of knowledge, temporal pressure, and dependence on others. For that reason, autonomy in medicine should not be described as isolated preference satisfaction. It is better understood as supported agency, exercised through communication, interpretation, trust, and professional guidance (Charles et al. 1997; O’Neill 2002; Entwistle et al. 2010; Beauchamp and Childress 2019). The late modern absolutization of autonomy can nevertheless enter medicine in consumerist form, especially within healthcare systems increasingly shaped by market logics and rising out of pocket costs. In this setting, the patient is recast as a chooser of services, while the clinician is recast as a risk managed provider whose task is to execute preference rather than to exercise clinical judgment. This weakens both patient and professional autonomy. Patients may be left alone with burdens of choice they cannot adequately process, and clinicians may retreat into defensive proceduralism rather than into fiduciary deliberation. Relational autonomy offers a more adequate model because it treats agency as socially scaffolded and responsive to vulnerability rather than as pure independence (Mackenzie and Stoljar 2000; Mol 2008; Montgomery 2023). Table 1 contrasts consumer or individual autonomy with relational autonomy, highlighting their different assumptions about the person, the clinician’s role, the institutional setting, the ideal of decision making, and the risks each model generates in clinical practice.

This distinction matters beyond narrower bioethical debates. Shared decision making is not a dilution of autonomy but one of the clearest settings in which autonomy appears as ethical maturity: patients require truthful information, clinicians require legitimate room for recommendation and warning, and both parties require a relationship capable of bearing uncertainty, disagreement, and responsibility over time.

**Table 1:** Contrasting models of autonomy in healthcare

Dimension	Consumer / individual autonomy	Relational autonomy
View of the person	Chooser imagined as self transparent and primarily preference bearing.	Agent understood as socially embedded, vulnerable, and interpretively dependent.
Role of clinician	Service provider who minimizes interference and liability.	Fiduciary guide who supports deliberation, understanding, and responsible choice.
Institutional layer	Contractual rights and personal choice; elective care / on-demand.	Standardised quality of care, within the management of scarce public resources.
Decision ideal	Preference execution once legal consent is documented.	Shared decision making sustained by dialogue, context, and trust.
Main risk	Isolation, defensive medicine, and burden shifted back to the patient.	Guidance without coercion, with responsibility distributed across the relationship.

### Identity, recognition, and platform punishment: from inclusion to punitive moralism

Recognition has become a central moral and political category in late modern democracies. Claims for recognition respond to real histories of misrecognition, exclusion, and structural inequality, and they have enabled important advances in inclusion, dignity, and institutional reform (Honneth and Honneth 1996; Fraser 2000; Fricker 2011). In this defensible form, recognition politics aims at equal standing within shared practices of justification, it seeks voice, repair, and access to institutions that confer rights and opportunities. Yet recognition can also shift from a civic demand for equal participation to a cultural demand for unconditional validation. When this shift occurs, identity is no longer a standpoint that enters deliberation, it becomes a moral boundary that limits deliberation. The public sphere is then reorganized around the protection of subjective integrity rather than the cultivation of common standards, and disagreement is easily recoded as negation or harm (Brown 2008; McNay 2008).

This section distinguishes three moral logics that are often conflated. First, recognition as equal respect, which supports democratic inclusion and can coexist with duties of reciprocity and civic restraint. Second, recognition as affective validation, where social interaction is governed by the imperative to affirm rather than to test reasons, and where conflict is framed primarily in psychological terms. Third, recognition as punitive moralism, where moral authority is asserted through sanction rather than argument, and where the primary political act becomes exposure, denunciation, and exclusion. The last two logics are intensified by therapeutic culture, which recodes moral conflict as injury and makes safety a dominant evaluative category, and by consumer individualism, which encourages a model of personhood defined by entitlement to satisfaction and control of discomfort (Rieff 1987; Illouz 2008; Sandel 2020).

Cancel culture is best understood within this broader transformation of moral economy. The term is contested and often used polemically. Here it refers to informal and networked punishment that is public, rapid, and frequently disproportionate, typically bypassing procedural standards and due process. It can target individuals in universities, workplaces, other institutions in the public sphere where collective pressure can be quickly mobilized and amplified, including social media and online platforms. It permeates fields such as medicine, journalism, politics and the arts, and it often operates through reputational damage, demands for exclusion, and institutional intimidation rather than through reasoned disagreement or formal complaint mechanisms (Norris 2023). This is distinct from legitimate accountability. Accountability is oriented toward evidence, proportionality, and reparation. Platform punishment is oriented toward visibility, alignment, and moral status within a group. It seeks not only to criticize an act but to mark a person as unfit for membership.

Digital platforms intensify these dynamics because they are designed around attention and engagement rather than deliberation. Their architectures reward speed, certainty, and affective intensity. Moral communication becomes compressed into signals of belonging, and public speech is reorganized as performance under conditions of constant surveillance (36–38). In this environment, denunciation can function as a low-cost display of moral competence, and punishment can be crowdsourced without stable standards. Algorithmic amplification further magnifies conflict by prioritizing content that produces outrage and spread rapidly, which in turn encourages categorical condemnation and reduces the space for revision, context, or forgiveness (Brady et al. 2017; Crockett 2017; Cinelli et al. 2021). The consequence is a shift in the function of moral language. Instead of enabling collective judgment, it becomes a weapon for boundary policing, a means of demonstrating purity and extracting deference.

The platform of public sphere also reshapes the status of vulnerability. Late modern cultures have rightly taken trauma, harm, and exclusion seriously. Yet vulnerability can become moral capital, a resource that confers authority through the exposure of suffering and that can narrow the space for contestation. This can correct epistemic injustice, but it can also produce a fragile discourse in which the capacity to endure disagreement is treated as oppression and where the burden of civic reciprocity is unevenly distributed (Illouz 2008; Fassin et al. 2009). In institutional settings, especially universities, workplaces, and professional organisations, reputational risk may then be managed through highly visible symbolic gestures, rapid distancing statements, or procedural overcorrection that signals moral alignment more than substantive adjudication. Studies of platform outrage, affective amplification, and institutional distrust help explain why such environments can make punishment feel unpredictable, contagious, and weakly connected to stable procedures, even when the original demand for recognition is legitimate (Marien and Hooghe 2011; Papacharissi 2015; Crockett 2017; Devine et al. 2021). We hypothesise that these dynamics weaken democratic life in several ways. First, they encourage autocensorship and conformity, since the costs of error are reputational rather than argumentative. Second, they replace deliberation with accusation and erode the distinction between critique and exclusion. Third, they undermine institutional trust, because institutions appear to act under pressure from online mobs, donors, or media cycles rather than under coherent standards and grounded deliberation. Fourth, they damage the conditions of moral development. A public culture that cannot tolerate contestation without moral panic makes it difficult to cultivate the virtues required for pluralism, including patience, humility, proportionality, and the capacity to revise one’s judgments.

A further institutional mechanism behind these dynamics is symbolic compliance. Under conditions of reputational volatility, universities, hospitals, and other organisations often respond to controversy by creating visible policies, statements, trainings, and committees that signal moral alignment while remaining weakly connected to everyday practice. Institutional theory described this as the decoupling of formal structure from operative life: organisations adopt procedures as public myths and ceremonies because legitimacy is at stake even when substantive transformation is limited (Meyer and Rowan 1977). From below, this makes punishment appear arbitrary. Institutions seem quick to align with public pressure, yet slow to clarify evidentiary thresholds, proportionality, or pathways of repair. Table 2 summarizes the difference between symbolic compliance and substantive engagement as two contrasting institutional responses to moral controversy.

**Table 2:** Two institutional responses to moral controversy

Dimension	Symbolic compliance	Substantive engagement
Primary aim	Visible legitimacy and rapid reputational protection.	Fact finding, proportionate response, and durable institutional learning.
Typical instruments	Statements, trainings, committees, and procedures loosely linked to practice.	Clear standards, resourced implementation, transparent investigation, and review.
Experience from below	Unpredictability, cynicism, and sense of theatre or procedural overcorrection.	Greater procedural trust, even when sanctions or criticism remain severe.

A critique of platform punishment is therefore not a defence of historical silencing or domination. It is a defence of democratic moral infrastructure. Recognition remains essential, but it must be reconnected to shared criteria of justification and to duties of civic restraint. Accountability remains necessary, but it must be linked to evidence, proportionality, and the possibility of restitution. Without these conditions, recognition can drift into coercive validation, and justice can be replaced by a punitive economy of exposure. This drift sets the stage for the further pathologies analysed as follows.

### Transgression without norms: adolescence, rebellion, and the formation of responsibility

Transgression in adolescence has often been understood as part of moral development because it stages an encounter between desire, prohibition, and the gradual internalisation of limits. Developmental and psychoanalytic traditions describe boundary testing as one route through which young people learn consequence, perspective taking, and responsibility, provided that limits are stable enough to be contested and interpreted rather than arbitrary or absent (Erikson 1994; O’Neill 2002; Allen 2010; Kroger et al. 2010; Winnicott 2018). In late modern contexts, however, transgression can lose this formative structure. Where norms are unstable, rapidly renegotiated, or displaced into platform performance, rebellion is more easily reduced to visibility, affective discharge, or identity signalling than to a practice through which judgment is consolidated. The result is a weaker transition from autonomy as choice to autonomy as answerability.

In psychoanalytic and developmental perspectives, norm formation depends on the child’s encounter with limits that are experienced as legitimate and consistent. Freud’s account of superego formation links prohibition and internalization to the structuring of conscience, where the subject learns that desire meets constraint and that actions have consequences that must be integrated rather than denied (Freud 2013). Winnicott describes the child’s exploration of limits within a holding environment, where boundary testing, if met with steady and non-humiliating containment, supports the gradual transition from dependence to self-regulation (Slochower 1991; Winnicott 2018). Erikson frames adolescence as a conflict between identity and role confusion, in which experimentation and rule testing are normal routes toward commitment, provided that the social world offers intelligible expectations and credible adult roles (Erikson 1994). Contemporary research supports this structure and that exploration often involves norm violation, and it can be developmentally productive when embedded in stable contexts that connect freedom to consequence and recognition to accountability (Kroger et al. 2010). When norms are inconsistent or purely negotiable, transgression can become chronic or nihilistic, failing to generate individuation or responsibility (Allen 2010; Fonagy et al. 2015).

The same logic applies at the level of democratic life. Dissent and civil disobedience presuppose a shared normative horizon against which rules can be contested. Transgression in this civic sense is not mere refusal. It is an appeal to higher principles and a demand that legitimacy be restored through public reasoning (Markovits 2005; Celikates 2016). Paradoxical though it sounds, dissent and civil disobedience take up an essential position within liberal democratic and human rights frameworks. Their justification emphasises dignity, autonomy and self-governance under the principle that certain state actions may be formally enacted, but morally unjust, calling for institutional protection and embedding these principles in international declarations and national legal frameworks.

Adolescence can be read as an initiation into this political structure, not simply by acquiring rights, but by learning how to contest norms without dissolving the possibility of common life. Empirical work on youth civic engagement suggests that norm challenging behaviour can express value clarification and moral commitment when it is linked to durable forms of participation and to institutions capable of transmitting standards of judgment (Kirshner 2007; Flanagan and Gallay 2014). Without such anchoring, rebellion is easily detached from public reason and reduced to expressive negativity or symbolic spectacle (Mounk 2018).

Late modern culture intensifies this detachment. In settings where authority is suspect, where standards are treated as oppressive, and where consumer individualism encourages the idea that discomfort is illegitimate, transgression loses its educative structure. Baudrillard’s argument that a world of permissiveness neutralizes the power of transgression captures part of this shift, as rebellion becomes aesthetic rather than consequential (Baudrillard 2009). Digital platform environments further accelerate the hollowing as they reward speed, intensity, and visibility, producing forms of outrage that are high in expression and low in commitment, and they compress moral conflict into signals of belonging rather than sustained practices of repair or institutional change (Dean 2005; Papacharissi 2015; Crockett 2017). Transgression becomes a currency within attention economies, and the costs that traditionally made dissent formative - risk, patience, conscientious involvement, personal consequence, and responsibility for outcomes - are often displaced by symbolic performance (Karpf 2017). This dynamic is visible in marketing and influencer culture, where provocation is commodified and controversy becomes a strategy rather than a moral act (Hearn 2008; Abidin 2015).

These transformations contribute to a crisis of adulthood understood as moral integration. Classical accounts linked adulthood with the capacity to sustain commitments, assume obligations, and occupy roles that bind the self to institutions and to others (Levinson 1986). Contemporary research describes a lengthened and ambiguous transition, with delayed milestones and weaker normative scripts, which affects not only economic trajectories but moral formation, since responsibility is less clearly rehearsed and socially recognized (Arnett 2000; Bynner 2005; Blatterer 2007 té 2014). In psychological terms this can produce reliance on external affirmation and difficulty sustaining norms that are not immediately rewarding or identity confirming (Twenge and Campbell 2008; Hendry and Kloep 2010). The subject remains capable of intense moral expression while struggling to convert expression into durable responsibility.

The core claim is therefore structural. Transgression is developmentally and civically productive only when it operates within a moral ecology that connects limits to meaning. Where norms are coherent, transgression can educate judgment and support the internalization of responsibility. Where norms are indeterminate, transgression becomes noise, and the passage from youthful rebellion to moral adulthood is interrupted. Rehabilitating transgression as an ethical and civic act requires restoring credible norms and practices, not to re impose repression, but to make responsibility learnable, contestable, and sustainable.

### Authority and the crisis of verticality: education, professions, and institutional trust

In liberal democratic societies, authority is frequently approached with suspicion. The experience of authoritarianism, ideological coercion, and institutional abuse has encouraged a cultural reflex in which verticality is treated as inherently oppressive. Yet the erosion of authority does not produce emancipation alone, if at all. It also produces disorientation, cynicism, and institutional paralysis as authority, properly understood, can avoid being domination. It is a symbolic and practical structure that enables orientation, transmits standards, and supports the intergenerational continuity without which freedom cannot be formed. The crisis of authority impacts education, family life, public institutions, and the professions and the weakening of verticality might undermine trust and destabilizes the ethical conditions of democratic life. Hannah Arendt’s analysis remains decisive because it distinguishes authority from both coercion and persuasion. Authority functions through recognition, it is neither forced by violence nor negotiated as mere opinion, and it depends on a shared belief that certain roles and traditions can legitimately guide action (Arendt 1993; Benhabib 2003). When tradition weakens and common reference points dissolve, authority loses its ground and the consequence is not simply a redistribution of power. A crisis of legitimacy might ensue where institutions struggle to justify guidance, where judgment becomes hesitant, and where responsibility is displaced into procedural risk management (or permanent negotiation).

Education offers a clear illustration. Anti-authoritarian impulses have often been translated into the flattening of pedagogical relations, generating role confusion between teacher and student and reluctance to set limits that are necessary for learning and psychosocial development (Masschelein and Simons 2002; Biesta 2015). Teaching is then reduced to facilitation, and authority is misread as repression rather than as the transmission of epistemic standards. Oakeshott’s account of education as initiation into a conversation clarifies what is lost. Authority in teaching is not primarily about imposing conclusions. It is about granting access to the language, disciplines, and criteria that make inquiry possible (Oakeshott 1991). When epistemic authority collapses, students are left with information without orientation and with expression without method, once again compounded by the cacophony of digital platforms. This collapse is intensified by consumer models of higher education, where institutions treat students as clients and where the demand for satisfaction can displace formative challenge and academic responsibility (Naidoo 2003; Molesworth et al. 2011; Lynch et al. 2012). Empirical work in teacher-student dynamics suggests that the withdrawal of clear authority, even when motivated by empowerment, can increase anxiety and disorganization, especially among adolescents who require structure to develop self-regulation and stable commitments (Hextall and Mahony 2013).

The crisis of authority extends beyond schools into civic institutions and public trust. Putnam’s diagnosis of declining civic association captures how weakened social membership reduces the legitimacy of institutions and the willingness to accept shared obligations (Putnam 2001). In fragmented societies, authority tends to polarize. It is either outsourced to technocratic expertise that appears distant and unaccountable, or replaced by celebrity and charisma that offer affective certainty without institutional responsibility (Hibbing and Theiss-Morse 2003; Papacharissi 2015). Research indicates that trust in government, public health authorities, and educational institutions has declined across many settings, with younger cohorts often exhibiting strong institutional scepticism alongside reliance on informal and unvetted sources of guidance (Marien and Hooghe 2011; Hetherington and Rudolph 2015; Devine et al. 2021). This combination produces epistemic fragmentation and vulnerability in the face of true oppression. The authority needed to coordinate collective action is weakened precisely when societies face problems that require shared compliance, long term planning, and civic restraint.

The professions reveal the same pattern in a sharper ethical register. Traditionally, professional authority rested on competence, role, and a vocation structured by internal goods and obligations. In late modern settings, professional authority is increasingly reframed as a service relationship governed by consumer expectations, procedural compliance, and reputational risk. In medicine and mental health, this shift can degrade clinical judgment. The professional is expected to guide yet is treated as morally suspect, and the encounter becomes organized around strict consent forms, liability waivers, protocols, and overall defensive documentation rather than around trust and responsibility (Montgomery 2023). Clinicians and teachers are placed in a double bind, as they must lead without being granted legitimacy to lead. Research on burnout and professional disillusionment suggests that the loss of vocational narrative and the rise of proceduralism contribute to moral injury and withdrawal, since responsibility is demanded while authority is delegitimized (Spickard Jr 2002) In this setting, institutional reliability is weakened from within, because professionals cannot sustain commitment when their role is reduced to transactional delivery.

A further driver of authority loss is the shifting moral economy of respect. Respect is increasingly grounded not in office or competence but in experience, particularly vulnerability, trauma, and marginalization. This shift has emancipatory aspects: it corrects exclusions and highlights epistemic injustice (Fricker 2011; Medina 2013). Yet when authority is awarded primarily through experienced suffering, institutional roles lose their normative neutrality and become sites of affective negotiation. Authority becomes contingent on emotional alignment rather than on publicly knowable standards, which increases polarization and undermines procedural legitimacy (Illouz 2008; Fassin et al. 2009; Lichterman and Eliasoph 2014). In classrooms, clinics, and public administration, guidance is then interpreted less as responsibility and more as domination, while disagreement is interpreted less as contestation and more as invalidation. The result is a weakened capacity to transmit norms and to educate judgment.

The crisis of verticality is therefore not simply a cultural mood. It is a structural problem for democratic ethics. Without legitimate authority, in the sense of a power collectively acknowledged and exercised within ethical, moral, and legal frameworks, norm formation weakens, learning becomes uncertain, and institutions cannot sustain trust. The task is not to restore authoritarianism. It is to rehabilitate authority as a formative and accountable structure, grounded in competence, role responsibility, fair procedures, and transparent standards, capable of guiding without coercing and of transmitting norms without demanding unconditional deference. Without such a reconstruction, both citizens and professionals remain suspended between fear of imposing and fear of being irrelevant, and democratic life loses one of its basic conditions of continuity and responsibility. This can also be described as a problem of institutional withdrawal. When authority is delegitimized, institutions do not disappear; instead, they increasingly offload interpretation, moral navigation, and risk management onto individuals while preserving managerial oversight. Subjects are then told to self author their norms in environments saturated by surveillance, competition, and opaque evaluation. The combination of maximal formal responsibility and minimal symbolic guidance helps explain why anxiety, distrust, and fragility rise together. Freedom is enlarged at the level of declaration while the practical conditions for bearing freedom are thinned (Hetherington and Rudolph 2015; Devine et al. 2021).

The link to fragile autonomy can be stated directly. Where legitimate asymmetry disappears, subjects must rely more heavily on peer recognition, immediate feedback, and self authored norms. This may enlarge formal choice, yet it weakens the stable external scaffolding through which frustration tolerance, trust, and durable responsibility are formed. In educational and professional settings, the absence of recognised authority can therefore intensify anxiety and moral uncertainty rather than reduce them, leaving subjects highly expressive yet poorly anchored in roles, standards, and intergenerational continuity (Erikson 1994; Illouz 2008; Hextall and Mahony 2013; Biesta 2015).

### The paralysis of opinion: from repression to hyperpluralism

Freedom of speech and opinion has long been a defining feature of liberal democracies, marking a rupture with regimes organized around censorship and enforced orthodoxy. Yet in late modern societies the status of opinion has become paradoxical. Formal protections remain robust, but the cultural and psychological space for sustained, divergent, and reasoned opinion appears to be contracting. The problem is less prohibition than distortion. Opinion is increasingly detached from deliberation and reconfigured as performance under conditions of saturation, polarization, and platform mediation. This section examines the shift from repression to expressive overload, and the way hyperpluralism can weaken judgment, civic courage, and the shared standards that make disagreement intelligible. Historically, opinion was constrained by external censorship, state power, religious authority, and institutional discipline. In many contemporary contexts, those constraints have receded, but they have been replaced by discursive saturation, an environment of constant expression that does not necessarily produce common understanding (Dean 2005; Wimmer 2015). The result is not a stable public sphere of dialogic reason but a proliferation of signals with limited mechanisms for convergence, revision, or mutual learning (Papacharissi 2002; Habermas 2006). Once again, digital platforms intensify this condition. Their attention economies reward brevity, immediacy, and affective intensity over context and justification (Freelon et al. 2020). Opinion therefore shifts from a reflective act to a rapid positioning. It becomes branding, alignment, and identity display rather than a reasoned contribution to common inquiry (Graham and Hajru 2011; Van Dijck and Poell 2013). Turkle’s account of diminished conversational depth captures part of the consequence, expression expands while reflection contracts, and the ability to hold complexity without immediate resolution weakens (Turkle 2011).

Hyperpluralism further destabilizes judgment. In settings where moral frameworks are fragmented and where disagreement is interpreted through affective lenses, strong opinions are experienced less as contestable claims and more as threats to belonging. The sacralization of identity and value conflict suggests that certain positions are treated as non-negotiable, making disagreement appear not as critique but as offense or negation. This is reinforced by affective polarization, where political affiliations become markers of moral character rather than provisional commitments open to revision (Druckman and Levendusky 2019; Iyengar et al. 2019). Under these conditions, opinion functions as a litmus test. It is less a judgment about the world than a signal of who one is and where one belongs. The psychological outcome is expressive inhibition – as the reluctance to speak in classrooms, workplaces, and professional contexts, not because speech is legally forbidden, but because the perceived social cost of misalignment is high and unpredictable. This produces a civic paradox: the norm to speak is strong, yet the conditions for measured, revisable discourse are weakened.

Platform dynamics compound this transformation by reorganizing public communication as exposure. Social media environment’s function more as stages than forums. Users curate opinions to secure status, protect group membership, and avoid reputational risk rather than to test arguments in good faith (Marwick and Boyd 2011; Wayne 2011). Algorithmic amplification encourages homophily and rapid escalation, increasing within group certainty and hostility across groups (Bail et al. 2018; Cinelli et al. 2021). Han’s description of exposure as vulnerability captures how speech becomes inseparable from self-presentation. The person is increasingly visible and increasingly afraid, which makes opinion feel risky and yet compels constant participation (Han 2018). This has consequences for political life. Research on digital deliberation suggests that affective contagion, speed, and moral certainty often outpace reasoned exchange, fueling polarization and distrust even when participation increases (Sydnor 2019; Guess et al. 2020).

One outcome is the disappearance of moderate, evolving, or ambiguous opinions. Hyperpluralism does not necessarily expand the space of nuance. It can produce a binary public culture in which opinion is either a performance of virtue or a performance of transgression, and in which the middle ground is socially costly (Grubbs et al. 2019). This is especially consequential in universities, journalism, and professional associations, institutions historically responsible for hosting plural inquiry and disciplined disagreement. Surveys and institutional reports indicate growing self-censorship and risk avoidance in such settings, driven less by state repression than by reputational pressures and internal norms (Georgetown University 2020; Knight Foundation and Ipsos 2022). As these institutions become more managerial and market sensitive, they may also prioritize conflict avoidance over intellectual formation, reinforcing the drift from judgment to safety and self-preservation. The contemporary crisis of opinion therefore stems less from its suppression than from its inflation and moralization. When public speech is saturated, identity coded, and platform amplified, opinion becomes both omnipresent and fragile. The democratic function of opinion, reasoned dissent, revisable judgment, and shared inquiry is displaced by alignment, anxiety, and performative certainty. Rehabilitating opinion as a civic and ethical practice requires more than formal free speech protections. It requires cultural and institutional norms that support practices of slow judgment, proportional response, interpretive charity, and the acceptance of contestation as a condition of plural democratic life.

### Rights without duties: from legal personhood to ethical erosion

The modern subject is often constructed through the grammar of rights, entitlements to freedom, dignity, security, and non-discrimination. Since the postwar expansion of the human rights frameworks and liberal democratic constitutions in the mid 1950s, rights language has become the dominant mode of articulating political and ethical claims. Yet, when rights become culturally primary while civic duties are treated as optional, merely contractual, or morally suspect, civic life loses part of its ethical scaffolding. Citizenship then risks being interpreted mainly as self-assertion rather than contribution, and democratic membership is reorganized around claims and entitlements without a parallel culture of responsibility. The ascendancy of the rights discourse is rooted in the liberal revolutions of the eighteenth century and codified, in the aftermath of World War II, in international frameworks such as the Universal Declaration of Human Rights, albeit the sovereignty of states continued being seen as a more enforceable normative (Moyn 2010). In the second half of the twentieth century it expanded beyond civil and political liberties to include social, economic, cultural, and identity based claims (Mutua 2008; Moyn 2010). This expansion has been necessary for the protection of marginalized groups and for the correction of structural exclusions. At the same time, multiple traditions have warned that a polity cannot be sustained by entitlements alone. Civic republican accounts emphasize participation and service, while communitarian critiques highlight the dependence of rights on shared moral norms and practices (Dagger 1997; Galston 2005; Etzioni 2013). Empirical work suggests that, when civic identity is framed primarily as entitlement, engagement may drift toward episodic claim making rather than stable institutional participation, particularly among younger cohorts socialized into individualized political repertoires (Dalton 2009; Norris 2023).

A clearer diagnosis requires specifying what is meant by duties, namely obligations owed by individuals to other persons, institutions, and shared worlds. These duties have three overlapping normative statuses. They are civic expectations internal to democratic membership, ethical obligations grounded in interpersonal and professional relations, and structural conditions for sustaining institutions, public trust, and long term common goods. They are therefore neither mere conventions nor simply private virtues. They form a taxonomy that corresponds to distinct domains of democratic life. First, civic duties concern membership of a political community. They include duties of participation, voting, jury service or military service where relevant, engagement in civic associations, and public minded restraint, including acceptance of legitimate outcomes and willingness to bear costs for collective goods (Putnam 2001; Galston 2005). Second, relational duties concern the ethical obligations that arise within family, friendship, and care relations, including reliability, reciprocity, and responsiveness to vulnerability, which cannot be reduced to rights claims without losing moral texture (Mol 2008; Tronto 2013). Third, professional duties concern role based obligations such as competence, integrity, confidentiality, and fiduciary responsibility, which sustain trust in medicine, education, law, and public service (Yates 1982; Montgomery 2023). Fourth, epistemic duties concern truth oriented conduct in public life, including duties to seek evidence, suspend judgment while doing so, interpret others charitably, revise beliefs when warranted, and avoid manipulation, all of which become crucial under conditions of polarization and platform amplification (Sunstein 1999; Habermas 2006). Fifth, intergenerational duties concern stewardship toward future persons, ecological limits, and long term risks, obligations that cannot be secured by individual rights alone because they demand anticipatory responsibility (Jonas 1984; Byskov 2021). These categories overlap, but together they clarify what is lost when duties are culturally devalued. The loss is not obedience. It is the disappearance of normative expectations that form character, sustain trust, and stabilize common projects. Table 3 proposes which different duties can be thought across ethical and civic registers and which institutional function they carry.

**Table 3:** Duties across ethical, civic, and institutional registers

Duty category	Ethical register	Civic register	Institutional function
Civic	Recognition of indebtedness to a political community and willingness to contribute.	Participation, restraint, and acceptance of legitimate procedures.	Supports democratic continuity and public trust.
Relational	Reliability, reciprocity, and response to vulnerability.	Builds the micro social fabric of solidarity.	Prevents care from being reduced to contract alone.
Professional	Fiduciary integrity toward patients, students, clients, and citizens.	Protects lay persons within knowledge asymmetries.	Sustains legitimacy of medicine, education, law, and public service.
Epistemic	Truth seeking, evidence sensitivity, and willingness to revise judgment.	Enables good faith disagreement in plural societies.	Counters manipulation, fragmentation, and platform distortion.
Intergenerational	Responsibility toward future persons and ecological limits.	Links present sacrifice to long term common goods.	Supports sustainability and anticipatory governance.

Paul Ricoeur’s ethical anthropology offers a framework for reconnecting rights and duties without reverting to authoritarian moralism. Rights and duties are co-constitutive because autonomy is meaningful only when exercised in relation to others. Responsibility is not an external burden added to freedom, it is the ethical condition that binds agency to the vulnerability of others through care, promise, and accountability (Ricœur 1990). This implies a conception of personhood that resists the fragmentation of rights into individualized claims detached from the practices that sustain mutual recognition and institutional trust (Brownsword 2009). Hans Jonas extends this argument to intergenerational and technological contexts. When the scale of action exceeds immediate horizons, responsibility must be anticipatory. Rights without future oriented duties risk legitimizing present satisfaction at the expense of long term viability, a problem central to ecological politics and biotechnological governance (Jonas 1984; Beckerman and Pasek 2004; Gough 2017). The weakening of duties is reinforced by consumer individualism and by institutional marketization. Duties, other than the legal obligations, responsibilities, and functions of the state to guarantee and enable individual rights and preferences, are increasingly interpreted as constraints on autonomy rather than as achievements of ethical maturity. Choice, comfort, and personalization become dominant values, and obligation is reframed as imposition (Sennett 2006; Bauman 2013). And this has identifiable civic mechanisms.

One such mechanism is the client model of institutional membership. In education, treating students as clients encourages the expectation of outcomes without a parallel expectation of effort, discipline, or respect for epistemic authority, which weakens resilience and undermines the formative function of schooling (Naidoo 2003; Ecclestone et al. 2009; Molesworth et al. 2011). A second mechanism is the contractualization of work. When obligations are experienced as purely transactional, vocational narratives weaken, mentorship declines, and institutions lose the internal commitment needed for trust and continuity. The language of disengagement, including quiet quitting, is one expression of this drift from contribution to minimum compliance (Reizer et al. 2023). A third mechanism is the consumerization of care. When healthcare is framed as service delivery to clients, responsibility for outcomes becomes asymmetrical, and the clinical relationship shifts from shared deliberation to entitlement plus complaint, eroding trust and increasing procedural defensiveness (Mol 2008; Tronto 2013; Montgomery 2023). The same can be said of novel technologies, digital services, and other areas pervaded by consumer mentality. A fourth mechanism is the substitution of rights claims for civic repair. When public disagreement is processed primarily as a rights violation rather than as a contest of reasons within common institutions, moral life is juridified, and the space for compromise, forgiveness, and proportional response narrows.

The core claim is therefore structural rather than moralizing. Rights require duties to become politically sustainable. Rights protect agency, duties discipline agency. When rights language dominates while duties are culturally discounted, personhood is refigured as claim making without reciprocal obligation, and citizenship is weakened as a practice of discerned participation and restraint. The moral architecture of democratic life depends on their mutual reinforcement. When that reciprocity fails, the subject becomes reactive, fragile, and disengaged, and institutions lose the trust and continuity required to govern plural societies.

### From fragile autonomy to ethical maturity: reconstructing the social subject

The ethical architecture of late modern liberal societies has increasingly elevated autonomy as the primary moral value. Yet the preceding analyses suggest a paradox, as autonomy framed mainly as self-determination and protection from constraint can produce subjects who are expressive yet fragile, rights conscious yet responsibility averse, and intensely concerned with recognition yet poorly equipped for sustained moral commitment. The task is therefore reconstructive. Autonomy should be reinterpreted not as sovereign self-will but as ethical maturity, a formed capacity to govern oneself in relation to others, to norms, and to institutions, with the ability to answer for one’s actions over time. This section proposes a post autonomous framework in which autonomy is integrated with responsibility, narrative identity, and practices that cultivate judgment. Charles Taylor’s account of the dialogical self offers a first pillar. Identity is not discovered in solitude but developed through recognition within moral horizons. To be a self is to orient oneself within frameworks of strong evaluation, not merely to choose among preferences (Taylor 1989). Recognition is therefore not equivalent to emotional validation. It is a moral relation that can sustain agency only when it includes accountability and shared meanings (Honneth and Honneth 1996; Ikäheimo and Laitinen 2011) Empirical work on narrative and developmental processes supports this point, coherent identity formation depends on interpersonal narrative scaffolding and socially mediated evaluation, not only on private authenticity (Fivush 2011; McLean and Syed 2015). A reconstructed autonomy must therefore be dialogical, requiring spaces where persons can be recognized without being insulated from disagreement, and where claims to identity can enter public reasoning without becoming moral vetoes.

The reconstructive proposal rests on a definite anthropology. The human person is understood here as relational, temporally extended, vulnerable, and capable of agency, but never self originating in a complete sense. Persons become autonomous through language, recognition, discipline, and participation in shared practices; they are narrative beings who acquire freedom through formation rather than possessing it as an unconditioned psychological fact. On this view, freedom, responsibility, and personhood are internally related. To act freely is not simply to express an inner state, it is to answer for oneself across time in ways that remain intelligible to others and sustainable within institutions (Taylor 1989; Ricœur 1990; Mackenzie and Stoljar 2000; MacIntyre 2007).

Narrative identity research reinforces this philosophical claim. Persons sustain agency by weaving past acts, present commitments, and anticipated futures into intelligible stories that can be revised without collapsing into fragmentation. Autonomy therefore depends on memory, promise, and the ability to own one’s actions diachronically rather than merely to intensify present preference. Developmental work on identity formation likewise suggests that maturity requires social recognition, interpretive scaffolding, and durable commitments, not simply expressive latitude (Fivush 2011; McLean and Syed 2015).

Emmanuel Levinas shifts the foundation further. Ethics begins not with the assertion of self-rule but with being addressed by the other. Responsibility precedes freedom, not as coercion but as the condition for moral seriousness (Levinas 1979; Bergo 1999). This does not abolish autonomy, it re situates autonomy as responsiveness. The mature subject is one who can be interrupted by the other and still remain capable of action. Moral psychology converges with this claim insofar as moral motivation is often driven less by abstract calculation rather than by attunement to vulnerability and suffering (Decety and Cowell 2014). In late modern cultures that interpret autonomy primarily as boundary protection, Levinas provides a corrective, ethical maturity framework that includes permeability, hospitality, and the acceptance of obligation that cannot be fully contractualized / that goes beyond contract (Kearney 2003; Gratton 2007). Paul Ricoeur bridges dialogical identity and ethical asymmetry through his notion of the capable human being. Autonomy is not mere volition. It is the capacity to act, speak, narrate, and assume responsibility through promise, memory, and accountability (Ricœur 1990). This view is crucial for reconstructing adulthood, regardless of fictional legal age thresholds. The mature subject is not the one who is maximally unconstrained. It is the one who can sustain commitments, acknowledge wrongdoing, and orient action toward future obligations. Ricoeur’s emphasis on narrative identity clarifies why autonomy collapses into fragility when it is detached from time. Where the self is forced into constant reinvention and permanent self-presentation, the ethical resources of continuity, promise, and forgiveness weaken. Autonomy as ethical maturity requires a temporal structure, a core self that can endure inconsistency, revise without disintegrating, and repair without abandoning responsibility.

Alasdair MacIntyre adds to this an institutional and virtue-oriented dimension. Moral agency cannot be formed in isolation. It requires participation in practices that have internal goods and standards of excellence, and it depends on traditions that transmit reasons across generations (MacIntyre 2007). Virtues are not preferences but stable dispositions cultivated through discipline, mentorship, and apprenticeship within institutions that embody normative expectations (Knight 2007). This has direct relevance to the earlier pathologies. If transgression becomes empty, authority delegitimized, opinion performative, and duties devalued, then the practices that form adults lose their formative power. A reconstructed autonomy therefore requires institutional settings capable of forming virtues, courage to dissent without nihilism, humility in judgment, fidelity to truth, and the willingness to carry obligations when they are inconvenient (Sanderse 2013; Arthur et al. 2016; Kristján Kristjánsson 2017). These perspectives converge on a single thesis. Ethical maturity is not the elimination of dependence but its transformation into responsibility. The adult is not the one who owes nothing. The adult is the one who can answer for commitments, tolerate disagreement without moral panic, and sustain obligations that bind freedom to common life. This reconstruction also clarifies what a post autonomous ethics should, and indeed, protect. It protects rights as necessary conditions of agency but refuses to treat rights as an exhaustive moral vocabulary. It protects recognition as a condition of dignity, but resists converting recognition into unconditional validation. It protects freedom of opinion but refuses to equate speech with branding and belonging. It protects autonomy but treats it as a formed capability rather than a default entitlement.

In education, autonomy as ethical maturity requires restoring the formative role of authority and standards, without returning to coercion. Schools and universities should treat learning as initiation into epistemic and moral practices, emphasizing disciplined inquiry, tolerance for uncertainty, and the ability to revise beliefs without humiliation. This includes explicit civic education in disagreement, proportionality, and responsibility, as well as pedagogies that combine recognition with demandingness rather than validation alone (Oakeshott 1991; Brighouse 1998; Biesta 2015). In the professions imparting knowledge, reconstruction requires rehabilitating professional authority as trustworthy judgment grounded in competence and fiduciary duty. Institutions should protect time for mentorship and apprenticeship, reduce purely procedural substitutes for trust, and restore vocational narratives that bind expertise to service and responsibility. Professional autonomy should be framed less as individual discretion and more as accountable practical wisdom exercised in relationship with patients, students, and publics (Tronto 2013; Montgomery 2023). In platform mediated publics, autonomy as ethical maturity requires environments that support deliberation, revision, and repair. This implies the push for design and governance norms that reduce incentive structures for outrage, accelerate context rather than virality, and create procedural pathways for accountability that are not primarily reputational punishments. It also implies cultural norms of digital restraint, including duties of interpretation, evidence, and proportional response that can counteract algorithmic polarization (Sunstein 1999; Papacharissi 2015; Tufekci 2015), holding also major private players accountable.

The medical context illustrates the point sharply. In clinical ethics, respect for autonomy must continue to protect informed consent, bodily integrity, and refusal of treatment, yet good care rarely consists in presenting neutral options to an isolated chooser. Patients often decide under conditions of illness, dependence, uncertainty, and unequal knowledge, which is why autonomy in medicine is better understood as supported, relational, and interpretive. Shared decision making requires trustworthy professional judgment, careful communication, and institutions that protect both patient voice and professional integrity; otherwise autonomy collapses either into paternalism or into consumerist abandonment (O’Neill 2002; Entwistle et al. 2010; Beauchamp and Childress 2019; Montgomery 2023).

Finally, civic rituals and associations must be renewed. Democracies depend on practices that make membership felt and duties intelligible, local associations, professional bodies, civic ceremonies, shared commemorations, and intergenerational narratives that connect freedom with responsibility (Ricœur 1990; Putnam 2001). Without such rituals of continuity, rights become isolated claims, and autonomy becomes a private project rather than a civic capacity. Autonomy can therefore be rescued from fragility by being re grounded in ethical maturity. This requires a reconstruction of subjectivity through dialogical recognition, responsibility to the other, narrative continuity, and participation in practices that form virtues. It also requires institutional reforms that make these capacities learnable. Education must form judgment, professions must rebuild trust, platforms must reduce punitive moralism and reward deliberation, and civic life must restore rituals and associations that bind rights to duties and freedom to responsibility.

### Limitations

This paper is a conceptual and interpretive review rather than an empirical study. It does not test causal hypotheses, quantify prevalence, or provide population level estimates of the phenomena discussed. The genealogy offered is selective and aims to clarify semantic shifts and institutional uptake, not to provide an exhaustive intellectual history of autonomy, authority, recognition, or duty. The interdisciplinary synthesis is purposive rather than systematic, and relevant literatures may have been omitted, especially work that frames therapeutic culture, recognition politics, or platform governance in more positive or context dependent terms. Empirical studies are used illustratively to constrain speculation, but they are not appraised through formal systematic review methods, and the evidentiary base varies across subtopics. Key constructs such as “cancel culture,” “authority,” and “duties” are contested and culturally variable, which limits generalizability across countries, political regimes, and institutional settings. Finally, the paper’s normative conclusions reflect a reconstructive philosophical stance and should be read as proposals for further debate and empirical investigation rather than as prescriptive policy recommendations.

## Conclusion

This essay has argued that autonomy remains a decisive achievement of modern moral and political life, yet it becomes unstable when treated as the whole of ethical life rather than as one element within a wider moral ecology. Tracing a genealogy from Kantian duty bound self legislation and Rousseauian civic self rule to liberal non interference and then to late modern expressive individualism and therapeutic culture, the paper has shown how autonomy can become detached from formative limits, shared standards of judgment, and the institutions that transmit responsibility. The result is a paradoxical subject who is formally protected and rhetorically empowered yet increasingly fragile, disoriented, and exposed to moral volatility. The implications are especially visible in medicine, where patient autonomy and professional autonomy are both weakened when autonomy is reduced either to consumer choice or to boundary protection alone.

Five linked pathologies were examined. Autonomy becomes a dominant moral grammar organized around entitlement and boundary protection. Transgression loses its developmental and civic function when norms are privatized or indeterminate. Opinion becomes performative in hyperpluralistic, platform mediated arenas, encouraging inhibition, polarization, and reputational fear. Symbolic authority erodes across education, professions, and public institutions, producing distrust and proceduralism. Duties are devalued in a rights centred moral economy, weakening civic cohesion and shared purpose. The paper proposes a reconstructive response in that autonomy should be redefined as ethical maturity, a formed capacity sustained by responsibility, legitimate authority, deliberative opinion, and duty. Institutional renewal in education, professional life, and digital publics is therefore central to restoring adult subjectivity and democratic resilience.
